# Burden of premature ventricular contractions beyond nonsustained ventricular tachycardia is related to the myocardial extracellular space expansion in patients with hypertrophic‐cardiomyopathy

**DOI:** 10.1002/clc.23445

**Published:** 2020-08-20

**Authors:** Hyemoon Chung, Chul‐Hwan Park, Yoonjung Kim, Jong‐Youn Kim, Pil‐Ki Min, Young Won Yoon, Kyung‐A Lee, Byoung Kwon Lee, Bum‐Kee Hong, Tae Hoon Kim, Se‐Joong Rim, Hyuck Moon Kwon, Eui‐Young Choi

**Affiliations:** ^1^ Division of Cardiology, Department of Internal Medicine Kyung Hee University School of Medicine Seoul South Korea; ^2^ Department of Radiology Gangnam Severance Hospital, Yonsei University College of Medicine Seoul South Korea; ^3^ Department of Laboratory Medicine Gangnam Severance Hospital, Yonsei University College of Medicine Seoul South Korea; ^4^ Division of Cardiology Gangnam Severance Hospital, Yonsei University College of Medicine Seoul South Korea

**Keywords:** hypertrophic‐cardiomyopathy, late gadolinium enhancement, myocardial fibrosis, premature ventricular contraction

## Abstract

**Background:**

Although nonsustained ventricular tachycardia (NSVT) is a risk factor for sudden cardiac death in hypertrophic‐cardiomyopathy (HCM), the impact of premature ventricular contraction (PVC) burden, in the absence of NSVT, is not well‐known.

**Hypothesis:**

PVC burden may be associated with myocardial fibrosis and genetic mutations in patients with HCM.

**Methods:**

Of the 212 patients prospectively enrolled to the HCM registry of genetics, 84 were evaluated with both cardiac magnetic resonance, 24‐hour Holter monitoring and genetic analysis. Among them, 71 patients have not been diagnosed with NSVT.

**Results:**

Patients with NSVT (n = 13) had a higher late gadolinium enhancement (LGE) amount, extracellular volume fraction (ECV), and prevalence of sarcomere mutations compared with patients without NSVT. Among patients without NSVT, those with LGE (n = 46) had a higher total PVC (109 ± 332 vs 7 ± 13, *P* = .003) and PVC burden (0.114 ± 0.225 vs 0.008 ± 0.014%, *P* = .003) during 24‐hour Holter monitoring compared with others. The %LGE and global ECV were correlated with PVC burden (r = 0.377, *P* = .001; r = 0.401, *P* = .001). The optimal cutoff value for PVC number for LGE was 45 (37.0% and 100% sensitivity and specificity, respectively) with 0.733 of the area under the receiver operating characteristic‐curve (*P* < .001). Thick filament gene mutation was more prevalent in the higher PVC burden group (41.2% vs 16.7%, *P* = .048).

**Conclusion:**

Total PVC burden is significantly related to increase in myocardial fibrosis in HCM patients without NSVT.

## 1 INTRODUCTION

Late gadolinium enhancement (LGE) represents myocardial fibrosis and is a well‐known sudden cardiac death (SCD) predictor in hypertrophic‐cardiomyopathy (HCM).^1^ Current guidelines suggest the presence of nonsustained ventricular tachycardia (NSVT) as a risk factor for SCD in patients with HCM.^2^, ^3^ However, there are some cases that NSVT is not detected in a 24 to 48 hour Holter monitoring, a recommendation of the current guidelines. A fibrotic myocardium could trigger and act as a substrate for both premature ventricular contraction (PVC) and NSVT.^4^ High‐PVC burden may reflect myocardial fibrosis even in the absence of NSVT. Moreover, sarcomere‐associated gene mutations have been associated with ventricular arrhythmia.^5^ We investigated (1) the association between NSVT and myocardial fibrotic burden, (2) the association between PVC burden and myocardial fibrotic burden in HCM patients without NSVT, and (3) contribution of sarcomere‐associated mutations for NSVT and PVC burden in a 24‐hour Holter monitoring.

## 2 METHODS

### 2.1 Study population

A total of 432 patients were enrolled in the HCM registry from 2006 to 2014 at our hospital. After excluding those patients who discontinued follow‐ups, were unable to visit the clinic due to migration or death, or were denied participation in the study, 212 patients were consecutively enrolled and underwent genetic testing. Of the 212 patients prospectively enrolled to the HCM registry of genetics,^6^ 84 were evaluated with both cardiac magnetic resonance and 24‐hour Holter monitoring. Among them, 71 patients (58 males, mean age: 71 ± 13 years) have not been diagnosed with NSVT. We divided the patients into two groups according to the presence of NSVT (NSVT group vs no NSVT group), then further divided the patients of “no NSVT group” into higher PVC burden group and lower PVC burden group according to the burden of PVC. The study protocol conforms to the ethical guidelines of the 1975 Declaration of Helsinki and was approved by our Institutional Review Board (3‐2015‐0019). A written informed consent was obtained for each subject.

### 2.2 24‐hour Holter monitoring analysis

A 24‐hour Holter monitoring was performed with SEER light (GE Healthcare) machine and was analyzed by both manual and automatic reviews of all 24‐hour echocardiography (ECGs). NSVT was defined as an episode of ventricular tachycardia (VT) with a heart rate of at least 120 beats per minute, lasting for at least three beats and persisting less than 30 seconds. Total PVC number was calculated, and two PVCs in a row were defined as couplet. PVC burden was defined as percent of total PVC number divided by total beats during monitoring.

### 2.3 Conventional echocardiography

A routine standard echocardiography study was performed to measure the systolic and diastolic parameters according to the current American Society of Echocardiography guidelines.^7^ Continuous wave Doppler was used to measure peak velocity across the LV outflow tract (LVOT), and the pressure gradient was calculated using the Bernoulli equation as follows: 4x (peak velocity across the LVOT).^.^ It was measured at resting and during Valsalva maneuver. LVOT obstruction was defined as a systolic pressure gradient ≥30 mmHg. Details are described in Supplementary Method S1.

### 2.4 Cardiac magnetic resonance imaging

Cardiac magnetic resonance (CMR) imaging was performed using a 1.5‐T scanner (Magnetom Avanto; Siemens Medical Solutions, Erlangen, Germany) with a phased array body coil. We analyzed LGE, T1, and extracellular volume fraction (ECV) for presenting myocardial fibrosis. The presence of LGE involvement in each segment and the total number of LGE‐involving segments was measured. Native T1 mapping with the modified Look‐Locker technique was performed during the mid‐diastolic phase, and post‐T1 mapping was performed 15 minutes after contrast media injection using the same slice axis and parameters as the pre‐T1 mapping. The percentage of LGE in LV mass was measured using dedicated quantitative analysis software (QmassMR 7.5 or 8.1, Medis, Leiden, Netherland) on LGE images with phase‐sensitive inversion recovery (PSIR). To improve the reproducibility, a radiologist and a cardiologist, both with more than 10 years of experience analyzed the LGE sizes. Boundaries of contrast‐enhanced areas were automatically traced in each short‐axis slice image. On LGE‐CMR images, the myocardium with an abnormal enhancement was defined as an area of hyperenhancement of more than five standard deviations from the remote myocardium. Remote myocardium was defined as nonenhanced myocardium, which is the opposite of hyperenhanced myocardium. Maximal signal was determined by computer‐assisted window thresholding of the enhanced area. Obvious artifacts, such as those caused by motion, were excluded using a tool from the software package. Total LGE volume was calculated by summing the LGE volumes of all the slices.^8^ Using the QMap RE (Medis, Leiden, Netherland), native T1, post‐T1, and ECV analyses were performed. The myocardial ECV was automatically calculated with the following equation:

ECV = (ΔR1 of myocardium∕ΔR1 of LV blood pool) × (1−hematocrit),

where R1 = 1∕T1 and ΔR1 = postcontrast R1−precontrast R1.^9^

The LGE, T1, and ECV values were measured according to the American Heart Association (AHA) 16‐segment model. Currently LGE, reflecting replacement fibrosis, is the most widely proven prognostic marker of SCD in HCM, therefore we chose LGE as a targeted value to determine the cutoff value of PVC burden.

### 2.5 Genetic analysis

#### 2.5.1 HCM gene panel design, DNA preparation, and data‐analysis of the HCM gene panel

A literature search of the PubMed database was performed for targeted gene selection for the comprehensive HCM‐specific panel. It included 82 genes (33 sarcomere‐associated protein genes, 5 phenocopy genes, and 44 nuclear genes linked to mitochondrial cardiomyopathy).^6^ The HCM genes consisted of 8 validated sarcomere genes and 25 putative HCM genes.^10^ We divided the sarcomere genes into two subgroups: thick filament genes (*MYH7*, *MYBPC3*, *MYH6*, and *MYL3*) and thin filament genes (other sarcomere genes except for thick filament genes). The details are described in Supplementary Method S2.

### 2.6 Statistical analysis

Continuous variables with normal distributions are reported as the mean ± SD or 95% confidence interval. The Student *t* tests were used to compare the means of the continuous variables that were approximately normally distributed between the two groups. Normality was determined using the Shapiro‐Wilk test. Categorical variables were reported as counts (or percentages) and were compared using the *χ* ^2^ tests. For the reproducibility test, paired sample *t*‐tests were performed and Pearson's correlation coefficients were calculated. Receiver operating characteristic (ROC) analysis was performed to identify the optimal cutoff values of burden of PVC for the presence of LGE. All clinical statistical analyses were performed using the SPSS version 25.0 statistical package (IBM Corp., Armonk, NewYork). A two‐sided *P*‐value <.05 was considered to be statistically significant.

## 3 RESULTS

### 3.1 Genetic, structural, and functional characteristics between patients with and without NSVT

We compared the baseline characteristics between patients with NSVT (n = 13) and without NSVT (n = 71) (Table 1). Patients with NSVT had more prevalent pathogenic or likely pathogenic sarcomere‐associated mutations (69.2% vs 26.8%, *P* = .005) and variants of uncertain significance (VUS) of sarcomere‐associated gene (84.6% vs 50.7%, *P* = .032). PVC couplet was more prevalent in the NSVT group compared with the no NSVT group (61.5% vs 21.4%, *P* = .006). In the echocardiographic analysis, the NSVT group had higher maximal wall thickness (22.3 ± 4.2 vs 18.9 ± 3.8 mm, *P* = .004), higher LV end‐systolic volume (31.8 ± 11.2 vs 24.3 ± 10.0 mL, *P* = .017), and lower LV ejection fraction (60.5 ± 9.3 vs 65.0 ± 5.7%, *P* = .023), e′ (4.3 ± 1.7 vs 5.4 ± 1.8 cm/s, *P* = .038), a′ (6.0 ± 1.5 vs 8.0 ± 1.7 cm/s, *P* = .001), and s′ (5.5 ± 1.6 vs 7.2 ± 1.7 cm/s, *P* = .001) compared with the no NSVT group. In the CMR analysis, the NSVT group had higher %LGE amount of LV (15.3 ± 13.2 vs 7.0 ± 8.3%, *P* = .004), higher number of LGE segments (5.9 ± 3.5 vs 3.1 ± 3.1, *P* = .003), and higher LV mass index (102.8 ± 24.0 vs 85.4 ± 25.3 g/m^2^, *P* = .024) compared with the no NSVT group.

**TABLE 1:** Comparison between patients with NSVT and without NSVT

	NSVT group (n = 13)	No NSVT group (n = 71)	*P* value
Age, years	57.4 ± 14.5	55.9 ± 13.0	.716
Male, n (%)	7 (53.8%)	58 (81.7%)	.038
Sarcomeric gene mutation, n (%)	9 (69.2%)	19 (26.8%)	.005
Sarcomeric gene variant including VUS, n (%)	11 (84.6%)	36 (50.7%)	.032
Thick filament mutation, n (%)	6 (46.2%)	16 (22.5%)	.092
Hypertension, n (%)	5 (38.5%)	41 (57.7%)	.236
Diabetes, n (%)	2 (15.4%)	14 (19.7%)	>.999
FHx of SCD‐first, n (%)	3 (23.1%)	4 (5.6%)	.071
FHx of SCD‐second, n (%)	2 (15.4%)	5 (7.0%)	.589
5 y SCD risk, %^*^	5.01 ± 2.97	1.72 ± 0.64	.002
AF, n (%)	5 (38.5%)	8 (11.3%)	.026
Total PVC beats, n	757 ± 865	73 ± 185	.015
Burden of PVC, n (%)	0.863 ± 1.044	0.078 ± 0.189	.019
The presence of PVC couplet, n (%)	8 (61.5%)	15 (21.4%)	.006
Beta blocker, n (%)	10 (76.7%)	50 (70.4%)	.749
Calcium channel blocker, n (%)	4 (30.8%)	23 (32.4%)	>.999
ACE inhibitor, n (%)	2 (15.4%)	3 (4.2%)	.169
ARB, n (%)	5 (38.5%)	33 (46.5%)	.764
Echocardiographic analysis			
Apical HCM, n (%)	2 (15.4%)	33 (46.5%)	.063
Maximal wall thickness, mm	22.3 ± 4.2	18.9 ± 3.8	.004
LVOT or mid‐LV obstruction, n (%)	4 (30.8%)	18 (25.4%)	.735
LV EDV, mL	82.6 ± 25.7	70.2 ± 22.9	.082
LV ESV, mL	31.8 ± 11.2	24.3 ± 10.0	.017
LAV, mL	73.3 ± 23.0	66.4 ± 24.1	.348
LAVI, mL/m^2^	43.0 ± 14.1	37.2 ± 14.5	.182
LVEF, %	60.5 ± 9.3	65.0 ± 5.7	.023
E, cm/s	70.3 ± 20.1	68.6 ± 17.5	.749
A, cm/s	63.6 ± 14.6	68.1 ± 19.9	.479
DT, ms	201.3 ± 44.4	210.9 ± 58.1	.575
e′, cm/s	4.3 ± 1.7	5.4 ± 1.8	.038
a′, cm/s	6.0 ± 1.5	8.0 ± 1.7	.001
s′, cm/s	5.5 ± 1.6	7.2 ± 1.7	.001
E/e′	18.0 ± 7.9	13.5 ± .4.9	.066
RVSP, mmHg	29.8 ± 8.2	28.3 ± 8.3	.561
CMR analysis			
LGE, n (%)	11 (84.6%)	46 (64.8%)	.208
% LGE amount of LV	15.3 ± 13.2	7.0 ± 8.3	.004
Number of LGE segments in LV	5.9 ± 3.5	3.1 ± 3.1	.003
T2‐average of 16‐setments, ms	56.5 ± 4.8	55.8 ± 3.1	.626
Native T1‐average of 16‐segments, ms	1035.8 ± 51.8	1022.3 ± 48.2	.365
Post T1‐average of 16‐segments, ms	586.7 ± 61.4	609.1 ± 63.5	.245
ECV‐average of 16‐segments, %	34.2 ± 5.1	32.1 ± 4.9	.158
LVMI, g/m^2^	102.8 ± 24.0	85.4 ± 25.3	.024
LV EDV, mL	134.1 ± 24.7	140.6 ± 29.2	.451
LV ESV, mL	51.5 ± 19.4	49.9 ± 19.9	.792
LVEF, %	62.1 ± 10.0	65.2 ± 8.6	.244

*Note*: Native and post‐T1 was measured in 67 patients due to poor image quality. ECV was measured in 65 patients after exclusion of six patients who were lack of hematocrit or T1 value.

Abbreviations: ACE, angiotensin converting enzyme; AF, atrial fibrillation; ARB, angiotensin receptor blocker; CMR, cardiac magnetic resonance; FHx, family history; ECV, extracellular volume; EDV, end‐diastolic volume; EF, ejection fraction; ESV, end‐systolic volume; GLS, global longitudinal strain; LAV, left atrial volume; LAVI, left atrial volume index; LGE, late gadolinium enhancement; LV, left ventricle; LVOT, left ventricular outflow tract; MR, mitral regurgitation; PVC, premature ventricular contractions; RVSP, right ventricular systolic pressure; SCD‐1, sudden cardiac death of first degree; SCD‐second, sudden cardiac death of second degree; VUS, variants of uncertain significance.

### 3.2 PVC burden and genetics according to the presence of LGE in patients without NSVT

We analyzed the characteristics of the patients without NSVT (n = 71). Baseline characteristics between these two groups according to the presence of LGE are compared in Supplemental Table S1. Patients with LGE had more prevalent sarcomere‐associated gene mutation (37.0% vs 8.0%, *P* = .011), thicker filament gene mutation (30.4% vs 8.0%, *P* = .039), and higher total beats (109 ± 332 vs 7 ± 13 beats per day, *P* = .003), PVC burden (0.114 ± 0.225 vs 0.008 ± 0.014%, *P* = .003) during a 24‐hour Holter monitoring compared with patients without LGE. In the echocardiographic analysis, patients with LGE had a lower a′ (7.7 ± 1.4 vs 8.6 ± 2.1 cm/s, *P* = .041) compared with patients without LGE. In the CMR analysis, the NSVT group had a lower post‐T1 (593.51 ± 56.93 vs 636.97 ± 66.29 ms, *P* = .006) and higher ECV (33.48 ± 4.80 vs 29.49 ± 3.89 ms, *P* = .001), LV mass (167.55 ± 52.90 vs 133.00 ± 40.96 g, *P* = .006), and LV mass index (92.45 ± 25.67 vs 72.29 ± 18.94 g/m^2^, *P* < .001) compared with patients without LGE.

### 3.3 Association between PVC and myocardial extracellular space expansion

**FIGURE 1:**
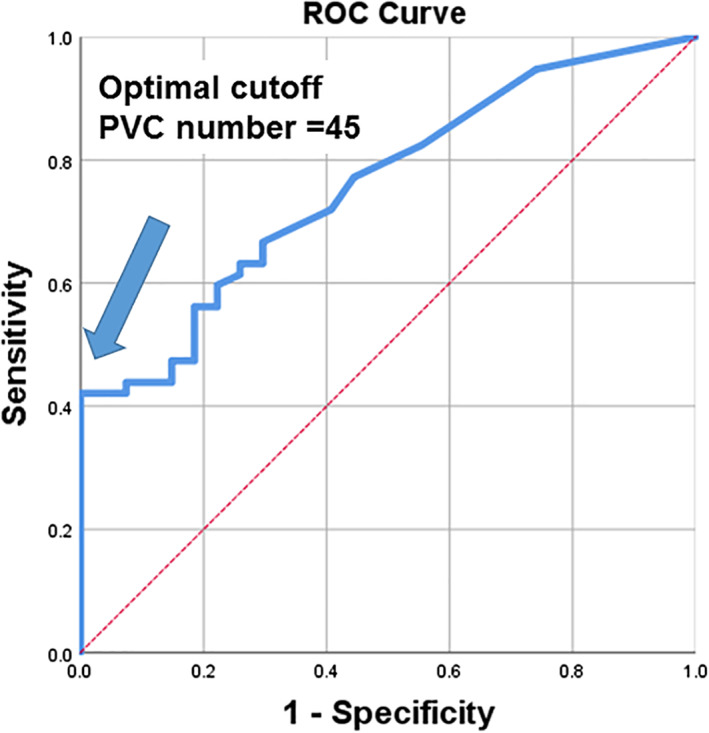
A receiver operating characteristic (ROC) curve for the presence of late gadolinium enhancement (LGE) of left ventricle. Area under the curve for LGE is 0.733 (95% confidential interval: 0.62 to 0.85, *P* = .001). The optimal cutoff value for premature ventricular complex number is 45 (37.0% and 100% sensitivity and specificity, respectively). PVC, premature ventricular contraction

The %LGE was correlated with total PVC beats (r = 0.358, *P* = .002) and PVC burden (r = 0.377, *P* = .001) of the 24‐hour Holter monitoring. Furthermore, E/e′ was correlated with %LGE (r = 0.263, *P* = .026). Moreover, ECV correlated with total PVC beats (r = 0.387, *P* = .001) and PVC burden (r = 0.401, *P* = .001). Post‐T1 tended to be correlated with PVC burden and total PVC beats (all *P* < .10). The optimal cutoff value for PVC number for the presence of LGE was 45 (37.0% and 100% sensitivity and specificity, respectively) with 0.733 of the area under the ROC curve (*P* = .001) (Figure 1).

### 3.4 Myocardial tissue characteristics and function between patients with higher and lower PVC burden

**FIGURE 2:**
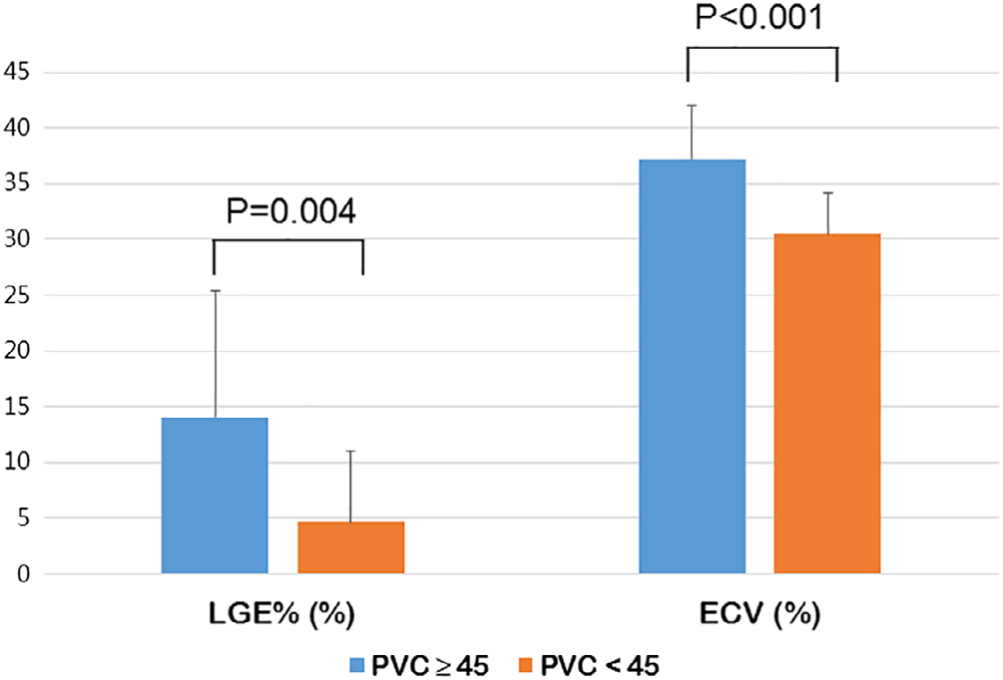
Comparisons of late gadolinium enhancement (LGE) amount and global extracellular volume fraction (ECV) between patients with higher premature ventricular complex (PVC) burden and lower PVC burden but without nonsustained ventricular tachycardia

When we divided the patients without NSVT into two groups according to PVC burden, the higher PVC burden group (total PVC ≥ 45, n = 17) had a tendency of more prevalent pathogenic or likely pathogenic sarcomere‐associated gene mutation (47.1% vs 20.4%, *P* = .056) although it was not statistically significant, and significantly higher prevalence of thick filament gene mutation (41.2% vs 16.7%, *P* = .048) compared with the lower PVC burden group (total PVC < 45, n = 54). In the CMR analysis, the higher PVC burden group had more prevalent presence of LGE (100% vs 53.7%, *P* = .001) and higher %LGE amount of LV (14.1 ± 11.3 vs 4.7 ± 6.1%, *P* = .004), number of LGE segments (4.5 ± 2.1 vs 2.6 ± 3.2, *P* = .022), post‐T1 (570.0 ± 62.9 vs 620.4 ± 59.6 ms, *P* = .006) and ECV (37.2 ± 4.8 vs 30.5 ± 3.7%, *P* < .001) compared with the lower PVC burden group. PVC couplet was more prevalent in the higher PVC burden group compared with the lower PVC burden group (47.1% vs 13.2%, *P* = .006) (Table 2 and Figure 2).

**TABLE 2:** Comparison between patients according to PVC burden

	Total PVC ≥ 45 (n = 17)	Total PVC < 45 (n = 54)	*P* value
Age, years	60 ± 12	55 ± 13	.133
Male, n (%)	15 (88.2%)	43 (79.6%)	.502
Sarcomeric gene mutation, n (%)	8 (47.1%)	11 (20.4%)	.056
Sarcomeric gene variant including VUS, n (%)	11 (30.6%)	25 (46.3%)	.267
Thick filament mutation, n (%)	7 (41.2%)	9 (16.7%)	.048
Hypertension, n (%)	10 (58.8%)	31 (57.4%)	>.999
Diabetes, n (%)	4 (23.5%)	10 (18.5%)	.730
FHx of SCD‐first, n (%)	1 (5.9%)	3 (5.6%)	>.999
FHx of SCD‐second, n (%)	0	5 (9.3%)	.328
5 y SCD risk, %^*^	1.63 ± 0.48	1.75 ± 0.68	.508
AF, n (%)	2 (11.8%)	6 (11.1%)	>.999
The presence of PVC couplet, n (%)	8 (47.1%)	7 (13.2%)	.006
Beta blocker, n (%)	10 (58.8%)	40 (74.1%)	.361
Calcium channel blocker, n (%)	8 (47.1%)	15 (27.8%)	.234
ACE inhibitor, n (%)	3 (17.6%)	0	.012
ARB, n (%)	10 (58.8%)	23 (42.6%)	.276
Echocardiographic analysis			
Apical HCM, n (%)	5 (29.4%)	28 (51.9%)	.163
Maximal wall thickness, mm	19.7 ± 5.0	18.6 ± 3.3	.418
LVOT or mid‐LV obstruction, n (%)	4 (23.5%)	14 (25.9%)	>.999
LV EDV, mL	68.6 ± 23.1	70.7 ± 23.0	.740
LV ESV, mL	25.9 ± 14.2	23.8 ± 8.4	.445
LAV, mL	75.6 ± 33.8	63.6 ± 19.7	.072
LAVI, mL/m^2^	42.3 ± 19.2	35.5 ± 12.5	.091
LVEF, %	62.8 ± 6.7	65.6 ± 5.2	.068
E, cm/s	66.9 ± 13.1	69.1 ± 18.7	.674
A, cm/s	73.6 ± 25.3	66.6 ± 18.1	.248
DT, ms	211.8 ± 72.4	210.6 ± 53.8	.942
e′, cm/s	4.7 ± 1.5	5.6 ± 1.8	.056
a′, cm/s	7.9 ± 1.7	8.1 ± 1.8	.758
s′, cm/s	7.0 ± 1.9	7.2 ± 1.7	.693
E/e′	15.1 ± 5.4	13.0 ± 4.6	.106
RVSP, mmHg	29.8 ± 8.1	27.8 ± 4.6	.404
CMR analysis			
LGE, n (%)	17 (100.0%)	29 (53.7%)	.001
% LGE amount of LV	14.1 ± 11.3	4.7 ± 6.1	.004
Number of LGE segments in LV	4.5 ± 2.1	2.6 ± 3.2	.022
T2‐average of 16‐setments, ms	55.9 ± 3.5	55.7 ± 3.0	.825
Native T1‐average of 16‐segments, ms	1030.8 ± 44.8	1019.8 ± 49.3	.443
Post T1‐average of 16‐segments, ms	570.0 ± 62.9	620.4 ± 59.6	.006
ECV‐average of 16‐segments, %	37.2 ± 4.8	30.5 ± 3.7	<.001
LVMI, g/m^2^	88.0 ± 19.8	84.5 ± 26.9	.626
LV EDV, mL	142.2 ± 33.3	140.1 ± 28.2	.799
LV ESV, mL	52.2 ± 30.3	49.2 ± 15.6	.593
LVEF, %	65.0 ± 10.3	65.2 ± 8.1	.926

Abbreviations: ACE, angiotensin converting enzyme; AF, atrial fibrillation; ARB, angiotensin receptor blocker; CMR, cardiac magnetic resonance; FHx, family history; ECV, extracellular volume; EDV, end‐diastolic volume; EF, ejection fraction; ESV, end‐systolic volume; GLS, global longitudinal strain; LAV, left atrial volume; LAVI, left atrial volume index; LGE, late gadolinium enhancement; LV, left ventricle; LVOT, left ventricular outflow tract; MR, mitral regurgitation; PVC, premature ventricular contractions; RVSP, right ventricular systolic pressure; SCD‐1, sudden cardiac death of first degree; SCD‐second, sudden cardiac death of second degree; VUS, variants of uncertain significance.

## 4 DISCUSSION

This study reports on major findings with regard to ventricular arrhythmia in HCM. First, patients with NSVT showed higher myocardial fibrosis burden compared with patients without NSVT. Second, after excluding patients who have been diagnosed with NSVT, higher PVC burden was associated with higher %LGE and ECV, which represent myocardial extracellular space expansion. Third, a pathogenic or probably pathogenic sarcomere‐associated mutation was associated with NSVT and higher PVC burden.

### 4.1 Contribution of PVC burden and myocardial fibrosis in HCM

Myocardial fibrosis is an NSVT substrate, and both are established risk factors for SCD in HCM. However, contributing factors to PVC has not yet been established in HCM. PVC arises from tissue with increased automaticity, and it is generally considered to be a benign phenomenon in cases without structural heart disease. However, a recent study reported that higher prevalence of VT was induced in PVC patients with LGE compared with patients without LGE during programmed ventricular stimulation.^11^ O'Mahony C et al. reported that 72% of VTs were triggered by PVC, especially late‐coupled PVC with a prematurity index of >0.5 in 84% of the cases. In their study, PVC burden on a 24‐hour Holter monitoring had a tendency to determine PVC initiated VT in HCM.^12^ Previous studies reported that frequent PVC was a predictor of future VT in postinfarction patients.^13^, ^14^ Therefore, although the dominant mechanism is unclear, we can suggest that frequent PVC may be associated with NSVT in HCM by triggered automaticity or re‐entry formation. However, a 24‐hour Holter monitoring cannot always catch the presence of NSVT; therefore, prolonged ECG monitoring is recommended despite the inconvenience in its performance.^15^ In addition, previous studies revealed that PVC burden was related with SCD in the general population.^16^, ^17^ Cipriani et al. reported that exercise‐induced PVC was related with LGE in young athletes.^18^ Therefore, PVC burden could be a potential risk factor for SCD in HCM patients without documented NSVT in a 24‐hour Holter monitoring. In our study, PVC couplet was more frequently seen in the NSVT and higher PVC burden groups compared with the no NSVT and lower PVC burden groups, respectively. This finding supports that individualized approach, such as close monitoring and strict medication (beta‐blocker), must be considered for patients with higher PVC burden. In this study, the cutoff value of total PVC number to predict LGE was relatively small at 45, which may be due to the higher prevalence of apical HCM, which is known as relatively benign phenotype in HCM, and high rate of beta‐blocker medication.

Myocardial fibrotic regions are nonconducting and nonexciting areas that increase the conduction heterogeneity of cardiac tissue. In HCM, myocyte disarray and myocardial fibrosis play a critical role in the arrhythmogenesis by promoting dispersion of electrical depolarization and repolarization, which results in the unidirectional conduction block and re‐entry. Both fibrosis, as an abnormal substrate, and ischemia or hypoxia, as a trigger ectopic beat or fatal ventricular arrhythmia, contribute to high arrhythmic propensity in HCM.^4^ Consistent with previous studies, higher myocardial fibrotic burden was seen in the NSVT group. Furthermore, higher LGE% and ECV and lower post‐T1 value were seen in the higher PVC burden group compared with the lower PVC burden group even in the patients without NSVT in our study.

### 4.2 Sarcomere mutations, PVC burden, and NSVT in HCM

Mutations in sarcomere genes could activate proliferative and profibrotic signals to produce pathogenic cardiac remodeling as a primary phenotype in HCM.^19^ Sarcomere‐associated gene mutation may be associated with ventricular arrhythmia due to its contribution to myocardial fibrosis, although the results of previous studies were controversial.^5^, ^20^ Our study results also show that patients with pathogenic sarcomere mutation had higher LGE amount and ECV and the patients with NSVT had more prevalent pathogenic or likely pathogenic mutation and VUS, and a tendency of more prevalent thick filament mutation in sarcomere genes compared with others. After exclusion of patients with NSVT, higher PVC burden group had a tendency of more prevalent pathogenic or likely pathogenic sarcomere‐associated mutation and significantly higher prevalence of thick filament mutation compared with the lower PVC burden group. It has not been clarified whether thick filament mutation is associated with higher rate of ventricular arrhythmia in HCM.^21^, ^22^ A recent meta‐analysis reported that myosin the *MYH7* mutation group showed the highest rate of ventricular arrhythmia compared with the *MYBPC3*, *TNNT2*, and *TNNI3* mutation group.^21^

## 5 LIMITATION

First, our study enrolled a relatively small number of patients. Second, the association between PVC burden and future event of SCD and clinical outcomes could not be established. A follow‐up study is needed to investigate the predictive value of current PVC burden to future events.

## 6 CONCLUSIONS

Total beats and PVC burden are significantly related to the increase in myocardial fibrosis in patients with HCM but without NSVT. In the absence of NSVT, high‐PVC burden may provide information for individualized therapy and follow‐up plan to prevent fatal ventricular arrhythmia in HCM. Moreover, the presence of sarcomere‐associated gene mutation may be also considered as one of the risk factors for ventricular arrhythmia. Further study is needed to investigate the direct relation between PVC burden and SCD in HCM.

## SOURCES OF FUNDING

This work was supported by the Basic Science Research Program through the National Research Foundation of Korea (NRF) funded by the Ministry of Education. (2014R1A1A2055872).

## CONFLICT OF INTEREST

None. The authors had full access to and take full responsibility for the integrity of the database. All authors have read and agreed to the manuscript as written.

## Supporting information


**Appendix** S1: Supporting Information
