# Supplementary material for: Burden of premature ventricular contractions beyond nonsustained ventricular tachycardia is related to the myocardial extracellular space expansion in patients with hypertrophic‐cardiomyopathy
Source: Clin Cardiol. 2020 Aug 20;43(11):1317–25. doi: 10.1002/clc.23445 (PMC7661646; doi:10.1002/clc.23445)
Supplement: Supplementary file 1 — Appendix S1: Supporting Information [file CLC-43-1317-s001.docx]

**Method S1. Conventional echocardiography**

A routine standard echocardiography study was performed to measure systolic and diastolic parameters as follows: LV end-diastolic volume and LV end-systolic volume (LVESV) were measured with the biplane Simpson’s method, and LV ejection fraction was calculated. Left atrial (LA) volume was measured at the end-systole by the ellipsoidal method, and LA volume index was calculated as LA volume/body surface area (BSA). Peak early (E) and late (A) diastolic mitral inflow velocities were measured in apical four-chamber view. Tissue Doppler interrogation was performed in the septal mitral annulus in apical four-chamber view, followed by measurement of the peak systolic mitral annulus velocity (s′) and early diastolic mitral annulus peak velocity (e′). The ratio of E/e′ was calculated. LV wall thickness was measured in all cross-sectional planes. Contrast echocardiography was performed in patients with a poorly defined LV border.

**Method S2. Genetic analysis**

***DNA preparation***

Genomic DNA was extracted from EDTA-treated whole blood samples by using a QIAamp DNA Blood Mini kit (Qiagen, Hilden, Germany) on a QIAcube automatic nucleic acid extrac­tion instrument (Qiagen) according to the manufacturer’s in­structions. The DNA samples were used to analyze mtDNA and the HCM gene panel (nDNA).

***Library construction and sequencing of the HCM gene panel***

Library construction and sequencing of the HCM gene panel were performed, as previously described. [^1^](#_ENREF_1)

***Data analysis of the HCM gene panel***

Data analysis of the HCM gene panel was performed, as previously described.[^1^](#_ENREF_1)

**Table S1. Comparison between LGE and non LGE group in patients without NSVT**

|  | **LGE group (n = 46)** | **Non-LGE group (n = 25)** | **P value** |
| --- | --- | --- | --- |
| **Age**, years | 57.3 ± 13.6 | 53.5 ± 11.6 | 0.244 |
| **Male**, n (%) | 38 (82.6%) | 20 (80.0%) | >0.999 |
| **Sarcemeric gene mutation**, n (%) | 17 (37.0%) | 2 (8.0%) | 0.011 |
| **Hypertension**, n (%) | 28 (60.9%) | 13 (52.0%) | 0.616 |
| **Diabetes**, n (%) | 9 (19.6%) | 5 (20.0%) | >0.999 |
| **FHx of SCD-1^st^**, n (%) | 1 (2.2%) | 3 (12.0%) | 0.122 |
| **FHx of SCD-2^nd^**, n (%) | 2 (4.3%) | 3 (12.0%) | 0.337 |
| **5-year SCD risk**, %^*^ | 1.69 ± 0.62 | 1.77 ± 0.67 | 0.628 |
| **Sarcomere mutation**, n (%) | 17 (37.0%) | 2 (8.0%) | 0.011 |
| **Sarcomeric gene variant including VUS**, n (%) | 26 (56.5%) | 10 (40.0%) | 0.220 |
| **Thick filament mutation**, n (%) | 14 (30.4%) | 2 (8.0%) | 0.039 |
| **AF, %** | 5 (10.9%) | 3 (12.0%) | >0.999 |
| **Total PVC beats** | 109 ± 332 | 7 ± 13 | 0.003 |
| **Burden of PVC, %** | 0.114 ± 0.225 | 0.008 ± 0.014 | 0.003 |
| **The presence of PVC couplet, %** | 13 (28.3%) | 2 (8.3%) | 0.069 |
| **Beta blocker**, n (%) | 33 (71.7%) | 17 (68.0%) | 0.789 |
| **Calcium channel blocker**, n (%) | 17 (37.0%) | 6 (24.0%) | 0.301 |
| **ACE inhibitor,** , n (%) | 3 (6.5%) | 0 | 0.306 |
| **ARB,** , n (%) | 22 (47.8%) | 11 (44.0%) | 0.807 |
| **Echocardiographic analysis** | | | |
| **Apical HCM**, n (%) | 18 (39.1%) | 15 (60.0%) | 0.135 |
| **Maximal wall thickness**, mm | 19.59 ± 3.87 | 17.58 ± 3.27 | 0.031 |
| **LVOT or mid-LV obstruction, n (%)** | 12 (26.1%) | 6 (24.0%) | >0.999 |
| **LV EDV**, ml | 71.2 ± 21.6 | 68.5 ± 25.4 | 0.649 |
| **LV ESV**, ml | 25.0 ± 10.5 | 23.0 ± 9.1 | 0.440 |
| **LAV**, ml | 61.5 ± 21.0 | 58.9 ± 21.2 | 0.629 |
| **LAVI**, ml/m^2^ | 34.6 ± 12.6 | 32.7 ± 13.4 | 0.554 |
| **LVEF**, % | 64.5 ± 6.3 | 65.8 ± 4.3 | 0.339 |
| **E**, cm/s | 67.4 ± 15.2 | 70.7 ± 21.2 | 0.499 |
| **DT**, ms | 213.1 ± 64.5 | 207.0 ± 45.5 | 0.676 |
| **e’**, cm/s | 5.1 ± 1.5 | 6.0 ± 4.6 | 0.051 |
| **a’**, cm/s | 7.7 ± 1.4 | 8.6 ± 2.1 | 0.041 |
| **s’**, cm/s | 7.1 ± 1.6 | 7.4 ± 2.0 | 0.570 |
| **E/e’** | 14.0 ± 4.6 | 12.6 ± 5.3 | 0.266 |
| **RVSP, mmHg** | 28.0 ±7.4 | 28.8 ± 10.0 | 0.700 |
| **CMR analysis** | | | |
| **% LGE amount of LV** | 10.78 ± 8.54 |  | |
| **Number of LGE segments in LV** | 4.72 ± 2.56 |  |  |
| **T2-average of 16-setments**, ms | 56.01 ± 2.81 | 55.36 ±3.60 | 0.424 |
| **Native T1-average of 16-segments**, ms | 1027.10 ± 43.35 | 1013.61 ± 55.90 | 0.313 |
| **Post T1-average of 16-segments**, ms | 593.51 ± 56.93 | 636.97 ± 66.29 | 0.006 |
| **ECV-average of 16-segments**, % | 33.48 ± 4.80 | 29.49 ± 3.89 | 0.001 |
| **LV mass**, g | 167.55 ± 52.90 | 133.00 ± 40.96 | 0.006 |
| **LVMI**, g/m^2^ | 92.45 ± 25.67 | 72.29 ± 18.94 | <0.001 |
| **LV EDV**, ml | 139.12 ± 29.08 | 143.39 ± 29.94 | 0.561 |
| **LV ESV**, ml | 49.91 ± 22.42 | 49.99 ± 14.64 | 0.988 |
| **LV EF**, % | 65.3 ± 9.0 | 65.0 ± 8.2 | 0.895 |

ACE, angiotensin converting enzyme; AF, atrial fibrillation; ARB, angiotensin receptor blocker; CMR, cardiac magnetic resonance; FHx, family history; ECV, extracellular volume; EDV, end-diastolic volume; EF, ejection fraction; ESV, end-systolic volume; LAV, left atrial volume; LAVI, left atrial volume index; LGE, late gadolinium enhancement; LV, left ventricle; LVOT, left ventricular outflow tract; MR, mitral regurgitation; NSVT, nonsustained ventricular tachycardia; PVC, premature ventricular contractions; RVSP, right ventricular systolic pressure; SCD-1^s^, sudden cardiac death of 1^st^ degree; SCD-2^nd^, sudden cardiac death of 2^nd^ degree.

Native and post-T1 was measured in 67 patients due to poor image quality.

ECV was measured in 65 patients after exclusion of 6 patients who were lack of hematocrit or T1 value.

**References**

1. Chung H, Kim Y, Park CH, et al. Genetic relevance and determinants of mitral leaflet size in hypertrophic cardiomyopathy. 2019;17(1):21.
